# High cell density and latent membrane protein 1 expression induce cleavage of the mixed lineage leukemia gene at 11q23 in nasopharyngeal carcinoma cell line

**DOI:** 10.1186/1423-0127-17-77

**Published:** 2010-09-22

**Authors:** Peter Han-Chung Yee, Sai-Peng Sim

**Affiliations:** 1 Faculty of Medicine and Health Sciences, Universiti Malaysia Sarawak, Lot 77, Seksyen 22 KTLD, Jalan Tun Ahmad Zaidi Adruce, 93150 Kuching, Sarawak, Malaysia

## Abstract

**Background:**

Nasopharyngeal carcinoma (NPC) is commonly found in Southern China and South East Asia. Epstein-Barr virus (EBV) infection is well associated with NPC and has been implicated in its pathogenesis. Moreover, various chromosome rearrangements were reported in NPC. However, the underlying mechanism of chromosome rearrangement remains unclear. Furthermore, the relationship between EBV and chromosome rearrangement with respect to the pathogenesis of NPC has not been established. We hypothesize that during virus- or stress-induced apoptosis, chromosomes are initially cleaved at the base of the chromatin loop domain structure. Upon DNA repair, cell may survive with rearranged chromosomes.

**Methods:**

In this study, cells were seeded at various densities to induce apoptosis. Genomic DNA extracted was processed for Southern hybridization. In order to investigate the role of EBV, especially the latent membrane protein 1 (LMP1), *LMP1 *gene was overexpressed in NPC cells and chromosome breaks were analyzed by inverse polymerase chain (IPCR) reaction.

**Results:**

Southern analysis revealed that high cell density resulted in cleavage of the mixed lineage leukemia (*MLL*) gene within the breakpoint cluster region (bcr). This high cell density-induced cleavage was significantly reduced by caspase inhibitor, Z-DEVD-FMK. Similarly, IPCR analysis showed that *LMP1 *expression enhanced cleavage of the *MLL *bcr. Breakpoint analysis revealed that these breaks occurred within the matrix attachment region/scaffold attachment region (MAR/SAR).

**Conclusions:**

Since *MLL *locates at 11q23, a common deletion site in NPC, our results suggest a possibility of stress- or virus-induced apoptosis in the initiation of chromosome rearrangements at 11q23. The breakpoint analysis results also support the role of chromatin structure in defining the site of chromosome rearrangement.

## Background

Nasopharyngeal carcinoma (NPC) is a common cancer in Asia, especially in Southern China and South East Asia [1]. NPC is well associated with chromosome rearrangements. Among them, chromosome gains are commonly found in 12p11.2-p12, 12q14-q21, 2q24-q31, 1q31-qter, 3q13, 1q13.3, 5q21, 6q14-q22, 7q21, 8q11.2-q23 and 18q12-qter. On the other hand, chromosome deletions are commonly found in 3p14-p21, 11q23-qter, 16q21-qter and 14q24-qter [2]. Much effort has been made to identify the candidate tumor suppressor genes and oncogenes, but studies investigating the mechanism(s) leading to the chromosome anomalies are rather lacking.

Epstein-Barr virus (EBV) is strongly associated with NPC [3] although the EBV genome is not required for epithelial to mesenchymal transition of NPC cells [4]. Nevertheless, various EBV antigens had been used in the diagnosis of NPC [5]. The actual mechanism of EBV infection contributing to carcinogenesis in NPC remains unclear. Nevertheless, EBV infection was found to induce apoptosis in neutrophills [6], and, overexpression of the EBV latent membrane protein 1 (*LMP1*) induced apoptosis in epithelial cells [7]. EBV infection also results in high molecular weight (HMW) DNA fragmentation [8] that is recognized as the initial chromosome breaks during early apoptosis [9]. HMW DNA fragmentation results from excision of chromosomal loops at their attachment sites to the nuclear scaffold via the matrix attachment region/scaffold attachment region (MAR/SAR) sequence [10]. Various enzymes including DNA topoisomerase II, caspase-activated DNase (CAD) and endonuclease G are involved in this chromosomal loop excision [10,11].

Apoptosis is a naturally occurring programmed cell death process, which can also be induced by a wide range of stimuli, including oxidative stress [12] and high cell density [13]. Initially apoptosis was thought to be an irreversible cell death process, however, there are emerging reports suggested that cells can survive apoptosis. These cells were shown to possess rearranged chromosomes [14,15] where the role of CAD was implicated [16]. Taken together the findings that EBV infection (as well as *LMP1 *expression) and stress induce or enhance apoptosis, while the apoptotic process may contribute to chromosome anomalies, it is possible that EBV infection-induced apoptosis may serve as a mechanism that leads to chromosome anomalies in NPC. Furthermore other virus has also been shown to induce chromosome aberrations in infected cells [17]. Therefore we hypothesize that during apoptosis induced by EBV infection or other apoptotic stimuli, chromosome breaks and rejoining occur at non-random sites. As a result, the surviving cells may harbor chromosome anomalies that are widely observed in NPC.

Any of the chromosome anomalies in NPC would first require the chromosome to break. To date, EBV or *LMP1*-induced apoptosis has not been reported to induce chromosome breaks within any specific gene. Therefore, in order to test our hypothesis, we induced NPC cells to undergo apoptosis followed by analysis of chromosome breaks within the mixed lineage leukemia (*MLL*) breakpoint cluster region (bcr). The *MLL *gene was chosen because: (1) *MLL *gene locates at 11q23 [18], which is a site commonly deleted in NPC [2], (2) *MLL *gene is commonly translocated in leukemia [19] and (3) *MLL *bcr contains MAR/SAR sequence [20].

In this report, we showed that both high cell density and *LMP1 *expression induced apoptosis in NPC cells and resulted in cleavage of the *MLL *bcr at the MAR/SAR region. This cleavage is mediated predominantly by CAD and partially by other nucleases.

## Methods

### Cell lines

NPC cell lines SUNE1 and HONE1, as well as the EBV genome-positive marmoset cell line, B95-8 (gifts from Prof. Dr. Choon-Kook Sam, National University of Singapore, Singapore) were cultured in RPMI 1640 medium supplemented with 10% heat-inactivated fetal bovine serum, L-glutamine (2 mM), penicillin (100 units/ml) and streptomycin (100 μg/ml), at 37°C with 5% CO_2_. The Epstein-Barr virus *LMP1 *recombinant plasmid was a generous gift from Dr. Eng-Lai Tan (International Medical University, Malaysia) and Prof. Dr. Choon-Kook Sam.

### Polymerase chain reaction (PCR) for digoxigenin (DIG)-labeled DNA probes synthesis

DIG-labeled DNA probe was synthesized using PCR Digoxigenin (DIG) Probe Synthesis Kit (Roche, Penzberg, Germany). The primers were MLL8005 5'-CCCTGAGTGCCTGGGACCAAACTAC-3' and MLL8342 5'-GGATCCACAGCTCTTACAGCGAACACAC-3'. pKS-MLLp (from Prof. Leroy Liu, USA), harboring a section of the *MLL *bcr was used as DNA template. Briefly, PCR reaction was carried out with 10 pg of DNA template, 50 pmol each of the primers, 200 μM each of dNTP, 1× of PCR buffer with 1.5 mM of MgCl_2 _and 2.6 U of ready to use enzyme mix in a total reaction volume of 50 μl. The initial denaturation step was carried out at 95°C for 5 min. This was followed by 30 cycles of denaturation at 94°C for 1 min, annealing at 60°C for 1 min and elongation at 72°C for 40 sec. A final elongation step of 72°C for 5 min was performed. The DNA probe synthesized detects the 3'-most 338 nucleotides of the *MLL *bcr, corresponding to nucleotides 8005-8342 of the *MLL *bcr [GenBank:U04737].

### Cell density-induced apoptosis and Southern analysis

Three 60 mm dishes were each seeded with 0.4 × 10^5^, 2 × 10^5 ^and 4 × 10^5 ^cells. In experiments where caspase inhibitor was used, cells were either treated with 50 μM of caspase-3 inhibitor II, Z-DEVD-FMK (Calbiochem, San Diego, CA) or the solvent DMSO. Cells were then allowed to grow for 4 days. Genomic DNA was extracted using Blood and Cell Culture DNA Mini Kit (QIAGEN, Hilden, Germany) following the manufacturer's protocol. Extracted genomic DNA was digested with 100 U of *Bam*H I (NEB, USA), followed by ethanol precipitation and analysis on 1% agarose gel together with the DIG-labeled DNA Molecular Weight Marker VII (Roche, Penzberg, Germany). Southern blotting was performed as described [21] except that 20× SSC was used. DIG-labeled DNA probe for Southern hybridization was synthesized as described above. Southern hybridization was performed using the DIG system and detection by DIG Luminescent Detection Kit (Roche, Penzberg, Germany) according to the manufacturer's protocol.

### Subcloning of LMP1 gene

The recombinant plasmid for *LMP1 *gene, pcDNA3.1/V5-His-TOPO-B95 (in short pcDNA-LMP1), was a generous gift from Prof. Choon-Kook Sam and Dr. Eng-Lai Tan. The *LMP1 *gene fragment was excised by *Kpn *I-*Xba *I (NEB, USA) digestion and subsequently subcloned into expression vector pTracer™-EF/V5-His B (in short pTracer) (Invitrogen, Carlsbad, USA). The resulting *LMP1 *recombinant plasmid is thus named pTracer-LMP1.

### Transfection of NPC cells with LMP1 expression plasmids

SUNE1 cells were seeded in RPMI medium without antibiotics and allowed to grow overnight to approximately 70% confluency in 60 mm culture dish. Transfection was carried out using LipofectAMINE™reagent and PLUS reagent (Invitrogen, Carlsbad, USA) following the manufacturer's protocol. Briefly, 2 μg each of the control vectors, pcDNA and pTracer; as well as the *LMP1 *expression plasmids, pcDNA-LMP1 and pTracer-LMP1 was individually diluted with serum free culture medium. PLUS reagent was then added to the plasmid DNA and the mixture was incubated at room temperature for 15 min to form the pre-complexed DNA. Separately, LipofectAMINE™reagent was also diluted with serum free culture medium and then combined with the pre-complexed DNA, followed by 15 min incubation at room temperature to form the DNA-PLUS-LipofectAMINE complex. Growth medium of the SUNE1 cells was then replaced with serum free culture medium, followed by addition of the DNA-PLUS-LipofectAMINE complex. The cells were then incubated at 37°C for 3 hrs in the presence of 5% CO_2_, followed by replacing the transfection medium with complete medium.

### SDS-PAGE and immunoblotting for detection of LMP1 expression

Transfected SUNE1 cells were harvested and washed with ice-cold phosphate buffered saline (PBS) followed by lysis in 2× SDS sample loading buffer [21]. Samples were boiled for 10 min, centrifuged, and equal volumes of the supernatant were analyzed on 10% SDS-polyacrylamide gel, followed by transfer onto Immobilon-P membrane (Millipore, Burlington, MA). Immunoblotting was performed with anti-V5 antibody (Invitrogen, Carlsbad, USA) at 1:1,000 dilution and S12 anti-LMP1 antibody (BD PharMingen, San Diego, CA) at 1:3,000 dilution. The blot was then exposed to SuperSignal^® ^West Pico Chemiluminescent Substrate (Pierce, Erembodegem, Belgium) followed by autoradiography. Lysate from the B95-8 marmoset cell was used as positive control.

### Nested inverse polymerase chain reaction (IPCR)

IPCR was carried out as described with modification [22]. Briefly, genomic DNA was extracted and digested with *Bam*H I (NEB, USA) at 37°C overnight. Klenow fill-in with DNA Polymerase I Large Fragment (NEB, USA) was performed, followed by cyclization by T4 DNA Ligase (NEB, USA) and subsequently linearization by *Msc *I (NEB, USA). Nested IPCR was performed with 200 ng of *Msc *I-digested template DNA, 10 pmol each of the primers, 200 μM each of the dNTP and 0.4 unit of Phusion polymerase (Finnzyme, Finland). PCR cycle condition was: 1 cycle at 98°C for 30 sec, followed by 30 cycles at 98°C for 10 sec, 61°C for 30 sec, 72°C for 15 sec and a final cycle at 72°C for 10 min. Second round PCR was performed with 2 μl of 5 time-diluted first round PCR products with similar cycle condition, except that the annealing and extension steps were carried out at 54°C for 30 sec and 72°C for 11 sec respectively. PCR products were analyzed on 1% agarose gel in 0.5× TBE buffer. The primers used were 5'-GCCAGTGGACTACTAAAACC-3' and 5'-CTTGTGGGTCAGCAATTCCTTC-3' in the first round, 5'-CTTCTATCTTCCCATGTTC-3' and 5'-TCCTCACTCACCTGATTC-3' in the second round.

## Results

### High cell density induces apoptosis and subsequent cleavage of the MLL breakpoint cluster region (bcr)

**Figure 1:**
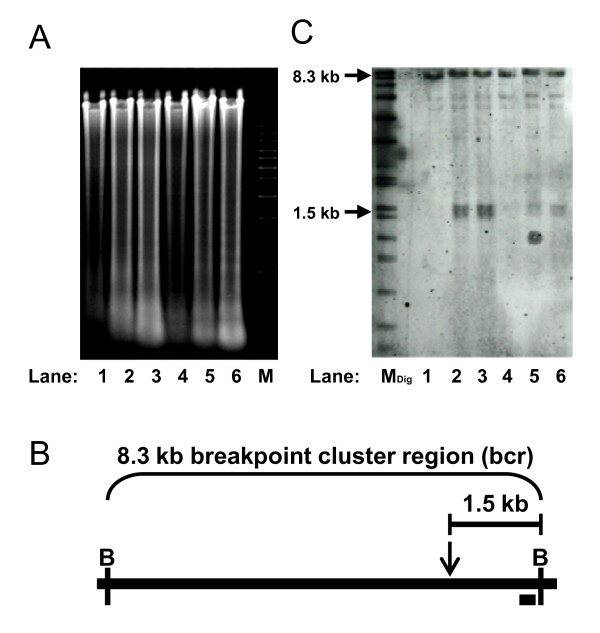
**High cell density induces apoptosis and subsequent cleavage of the *MLL *bcr**. (A) Ethidium bromide-stained agarose gel. SUNE1 (lanes 1-3) and HONE1 (lanes 4-6) seeded at cell number of 0.4 × 10^5 ^(lanes 1 and 4), 2 × 10^5 ^(lanes 2 and 5) and 4 × 10^5 ^(lanes 3 and 6) were harvested for genomic DNA extraction after 4 days of growth. DNA was digested with *Bam*H I and analyzed on 1% agarose gel. M represents the 1 kb DNA marker. (B) A schematic diagram illustrating the 8.3 kb *MLL *breakpoint cluster region (bcr). B represents the *Bam*H I restriction site. Black box indicates the position of the DNA probe and down arrow shows the anticipated site of DNA cleavage. (C) Southern hybridization analysis. Southern hybridization was performed using the DNA probe shown in (B). Arrows labeled 8.3 kb and 1.5 kb show the positions of the intact and the cleaved *MLL *bcr respectively. M_Dig _represents the DIG-labeled DNA marker (Roche, Penzberg, Germany).

To investigate the role of high cell density-induced apoptosis in causing chromosome cleavage, SUNE1 and HONE1 NPC cells were seeded at different densities and allowed to grow for 4 days. Genomic DNA was extracted, digested and analyzed on agarose gel. As shown in Fig. 1A, accumulation of small fragments of DNA was observed in cells seeded at higher density (lanes 2, 3, 5 and 6) as compared to those seeded at lower density (lanes 1 and 4). Subsequently, Southern hybridization was performed using a DNA probe hybridizing to the telomeric end of the *MLL *bcr as shown in Fig. 1B. This probe detects the 8.3 kb intact *MLL *bcr encompassed by the *Bam*H I restriction sites. Any cleavage within the *MLL *bcr, centromeric to the probe will be detected as fragments smaller than 8.3 kb. For both SUNE1 and HONE1 cell lines, a 1.5 kb fragment was detected at high cell density in addition to the 8.3 kb intact *MLL *bcr, (Fig. 1C, lanes 2, 3, 5 and 6). This indicates that high cell density-induced apoptosis results in chromosome break within the *MLL *bcr 1.5 kb from the telomeric end (Fig. 1B).

### Caspase inhibitor reduces high cell density-induced MLL bcr cleavage

**Figure 2:**
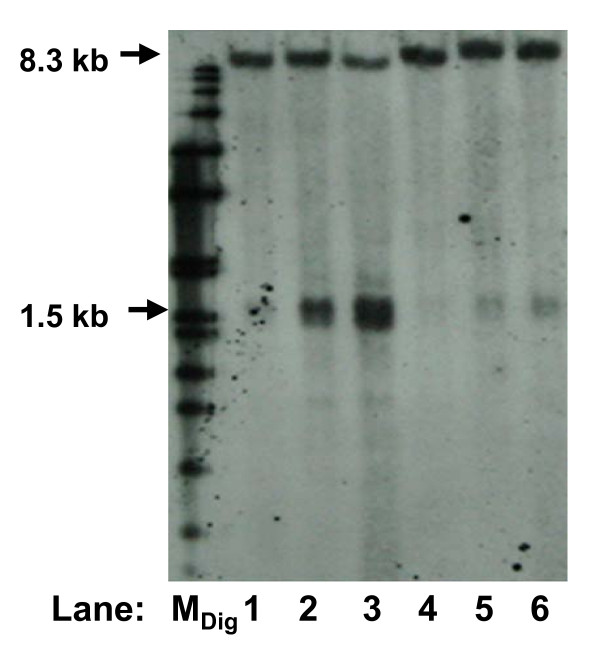
**Caspase inhibitor reduces high cell density-induced *MLL *bcr cleavage**. SUNE1 cell seeded at cell number of 0.4 × 10^5 ^(lanes 1 and 4), 2 × 10^5 ^(lanes 2 and 5) and 4 × 10^5 ^(lanes 3 and 6) were allowed to grow for 4 days in the absence (lanes 1-3) or presence (lanes 4-6) of 50 μM caspase-3 inhibitor II (Z-DEVD-FMK). Extracted genomic DNA was processed for Southern hybridization as described in methods. Arrows labeled 8.3 kb and 1.5 kb show the positions of the intact and the cleaved *MLL *bcr respectively. M_Dig _represents the DIG-labeled DNA marker.

The apoptotic nuclease, caspase-activated DNase (CAD) exists as a complex with the inhibitor of CAD (ICAD) [23]. During apoptosis induction, caspase cascade is activated, where caspase-3 cleaves ICAD, thus releasing the activated CAD [24]. Therefore, to investigate if the apoptotic nuclease is involved in cleavage of the *MLL *bcr during high cell density-induced apoptosis in NPC cells, caspase inhibitor was used to inhibit CAD. Consistent with the observation shown in Fig. 1C, high cell density resulted in cleavage of the *MLL *bcr, evidenced by the presence of the 1.5 kb fragment (Fig. 2, lanes 2 and 3). This cleavage was significantly reduced in cells treated with caspase inhibitor (Fig. 2, lanes 5 and 6). This finding suggests that CAD is involved in cleavage of the *MLL *bcr resulted from high cell density-induced apoptosis.

### Expression of LMP1 gene induces apoptosis in SUNE1 cells

**Figure 3:**
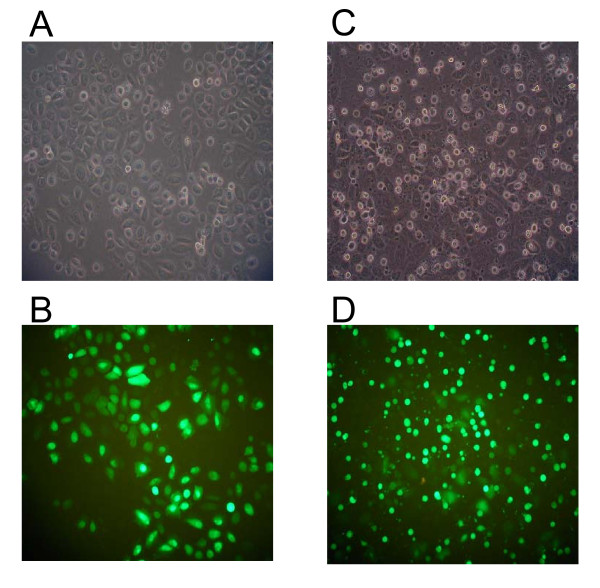
**Transfection of SUNE1 cell with *LMP1 *induces apoptotic cell death**. SUNE1 cells were transiently transfected with pTracer vector (A and B) or *LMP1 *expression plasmid, pTracer-LMP1 (C and D). Cell morphology was monitored under bright-field microscopy (A and C) as well as dark field microscopy (B and D). Expression of the green fluorescence protein, GFP, is observed as green colored cells.

In order to investigate the relationship between EBV, apoptosis and chromosomal rearrangements in NPC, SUNE1 cells were transfected with either the vector or the *LMP1 *expression plasmid to assess apoptosis induction and *MLL *bcr cleavage. Two expression plasmids were used, namely pcDNA and pTracer. Since the green fluorescent protein (*GFP*) gene and the *LMP1 *gene are on the same plasmid vector (pTracer), the expression of *GFP *is indicative of plasmid uptake by the cells and most likely the expression of *LMP1 *as well. As shown in Fig. 3A, pTracer-transfected cells showed normal morphology. Cells expressing *GFP *retained the normal morphology as well (Fig. 3B). By contrast, cells transfected with *LMP1 *expression plasmid showed the typical apoptotic morphology (Fig. 3C). Moreover, most of the *GFP*-expressing cells were found to be floating and blebbing, with a smaller population of *GFP*-expressing cells remained attaching to the flask (Fig. 3D). These results suggest that expression of *LMP1 *induces apoptosis in SUNE1 cells. Cells transfected with the pcDNA set of plasmids showed similar results under bright field microscopy (data not shown). Dark-field microscopy result is not available for the pcDNA set as it does not carry the *GFP *gene.

### Expression of LMP1 gene induces DNA breaks within the MLL bcr

**Figure 4:**
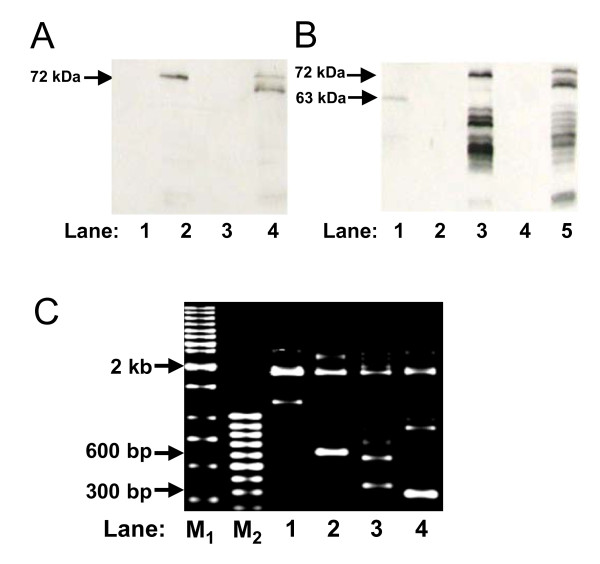
***LMP1 *expression induces cleavage of the *MLL *bcr**. (A) Detection with anti-V5 antibody. SUNE1 cells were either transfected with vectors, pcDNA or pTracer (lanes 1 and 3); or *LMP1 *expression plasmids, pcDNA-LMP1 or pTracer-LMP1 (lanes 2 and 4). Cell lysate was analyzed on 10% SDS PAGE, and *LMP1 *expression was detected by anti-V5 antibody. (B) Detection with S12 anti-*LMP1 *antibody. SUNE1 cells were either transfected with vectors, pcDNA or pTracer (lanes 2 and 4); or *LMP1 *expression plasmids, pcDNA-LMP1 or pTracer-LMP1 (lanes 3 and 5). Cell lysate was analyzed on 10% SDS PAGE, and *LMP1 *expression was detected by S12 anti-LMP1 antibody. Lysate from the EBV-positive B95 cell line was included as positive control (lane 1). Arrows labeled 72 kDa and 63 kDa indicate the size of the expressed LMP1 (with V5 epitope and His-tag) and the endogenous LMP1 of B95-8 respectively. (C) Detection of *MLL *bcr cleavage by IPCR. SUNE1 cells transfected with vectors, pcDNA or pTracer (lanes 1 and 3); or *LMP1 *expression plasmids, pcDNA-LMP1 or pTracer-LMP1 (lanes 2 and 4) were collected for genomic DNA extraction. DNA was processed for nested IPCR as described in methods. Arrow labeled 2 kb indicates the position of the IPCR product of the intact *MLL *bcr. Arrows labeled 600 bp and 300 bp indicate the positions of the IPCR products of the cleaved *MLL *bcr. M_1 _and M_2 _represent the 1 kb and 100 bp DNA marker respectively.

Expression of *LMP1 *gene was confirmed by Western blotting using anti-V5 (Fig. 4A) and S12 anti-LMP1 antibody (Fig. 4B). Expression was demonstrated in LMP1 transfectants (Fig. 4A, lanes 2 and 4; Fig 4B, lanes 3 and 5) as compared to the controls (Fig. 4A, lanes 1 and 3; Fig 4B, lanes 2 and 4). EBV-positive B95 cell was included as a positive control and the reported 63 kDa LMP1 protein was detected (Fig. 4B, lane 1). The discrepancy in the protein size observed (72 kDa in transfected cells and 63 kDa in B95-8 cell) is due to the reason that LMP1 was expressed in fusion with V5 epitope and His-tag in the transfected cells. In addition, multiple bands of possibly degraded LMP1 were also detected in these cells (Fig. 4B, lanes 3 and 5).

Subsequent to the observation of apoptotic morphology in *LMP1*-transfected cells, we intended to test whether expression of *LMP1 *results in cleavage of the *MLL *bcr by nested IPCR. As shown in Fig. 4C, both the vector-transfected and *LMP1*-transfected cells demonstrated the presence of a 2 kb band, which was derived from the intact *MLL *gene (Fig. 4C, lanes 1-4). Interestingly, cells transfected with the vectors (Fig. 4C, lanes 1 and 3) showed faint bands of sizes of less than 2 kb. From our experience, these bands might be contributed by those cells that were dying naturally while in culture as well as during the transfection process. On the other hand, cells transfected with *LMP1 *expression plasmids (Fig. 4C, lanes 2 and 4) showed very distinct and intense bands of sizes smaller than 2 kb. DNA sequencing of these bands (600 bp and 300 bp IPCR products recovered from Fig. 4C lanes 2 and 4 respectively) confirmed that they were the result of DNA cleavage within the *MLL *bcr. The precise breakpoints of the 600 bp and 300 bp were mapped to coordinates 7215 and 6782 respectively [GenBank:U04737]. These results suggest that expression of *LMP1 *induces apoptosis in NPC cells, and subsequently results in cleavage of the *MLL *bcr.

## Discussion

The association of EBV with NPC is well documented [3], and various chromosome anomalies are well reported in NPC [2]. However, the actual role of EBV in the pathogenesis of NPC is unclear and EBV's involvement in chromosome rearrangements remains to be elucidated. Other virus has been shown to induce chromosome aberrations in infected cells [17]. Similarly, *LMP1 *expression was found to induce aneuploidy in human epithelial cells [25]. Knowing that EBV infection and *LMP1 *expression induce apoptosis in mammalian cells [6,7], we wanted to answer a further question: is EBV-induced apoptosis a mechanism of chromosome rearrangement in NPC? Here, our results for the first time show that *LMP1 *expression and high cell density induce apoptosis in NPC cells and subsequently result in enhanced DNA cleavage within the *MLL *bcr at 11q23, a common chromosome deletion site in NPC.

It is important to note that, the breakpoints identified in this study fall within the bcr of the *MLL *gene. Cleavage of the *MLL *bcr has been extensively studied in leukemic cells, relating to chromosome translocation mechanism involving topoisomerase II [26] and apoptotic nuclease [14,21]. However, this is the first demonstration of apoptosis-induced cleavage of the *MLL *bcr in NPC cells. Since the *MLL *gene locates at 11q23 [18], a common chromosome deletion site in NPC [2], our findings support the possibility that chromosome deletion at 11q23 in NPC could begin at the *MLL *gene.

In our study, treatment with caspase inhibitor significantly reduced the *MLL *bcr cleavage. This parallels the observations in leukemic cells, suggesting the involvement of a caspase-dependent apoptotic nuclease [21], possibly the caspase-activated DNase (CAD) [23]. CAD associates with the nuclear matrix of apoptotic cells [27], facilitating its role in cleaving the base of the chromatin loops at the nuclear matrix or scaffold, generating high molecular weight (HMW) DNA during early stage apoptosis [28]. CAD was also shown to cause DNA fragmentation producing the characteristic nucleosomal DNA ladder [23]. However, CAD is not the sole enzyme for DNA cleavage at nuclear matrix, as it was found to be dispensable for HMW DNA fragmentation during early stage apoptosis in chicken DT40 cells [29]. This observation tallies with our result that caspase inhibitor did not abolish the *MLL *cleavage completely, suggesting the possible involvement of other nucleases. One promising candidate is endonuclease G (Endo G) [11], which is one of the effectors of caspase-independent cell death pathway [30]. Interestingly, both CAD and Endo G preferentially cleave DNA at the internucleosomal linker DNA. They also cleave at the borders of chromatin loops, releasing chromatin domains of sizes ≥ 50 kb [11]. This chromatin loop domain structure is maintained by the interaction of specific sequences known as the matrix attachment region/scaffold attachment region (MAR/SAR), with the nuclear matrix proteins [31]. During early apoptosis, genomic DNA is cleaved at the base of the chromatin loop, results in the formation of HMW DNA of 50 - 300 kb [32].

In this study, the *MLL *cleavage sites observed in the NPC cells localized within the MAR/SAR sequence of the *MLL *bcr [20], suggesting that both CAD and Endo G could be involved in introducing the breaks during early apoptosis. This is a very crucial observation as we hypothesize that during apoptosis, the genomic DNA is being cleaved at the base of the loop, and rejoined erroneously upon the cell's attempted repair. As a result, cells that survive the apoptotic process may harbor various kinds of chromosome anomalies. Logically, only those cells that are at the early stage of apoptosis can be rescued and survive apoptosis.

In addition to CAD and Endo G, DNA topoisomerase II is another important player in the excision of the chromatin loops during early apoptosis [33]. Poisoning of topoisomerase II by etoposide and oxidative stress resulted in chromatin loop excision [10,33]. This is entirely logical as topoisomerase II is one of the two major proteins found in the nuclear scaffold [34]. Interestingly, CAD interacts with topoisomerase II and enhances topoisomerase II's decatenation activity *in vitro *[35]. Since EBV infection introduces oxidative stress to the cell [36], thus our results of *MLL *bcr cleavage could be partly mediated by topoisomerase II and Endo G in addition to CAD.

Conventionally, apoptosis is known to be an irreversible programmed cell death process [37]. However, some of the cells can survive apoptosis. These cells may harbor rearranged chromosomes that contribute to leukemogenesis [15]. This is supported by the observation that apoptotic triggers resulted in the formation of *MLL-AF9 *fusion gene in leukemic cells that are capable of division [14]. Although various mechanisms have been proposed, chromatin structures at the breakpoint cluster regions were recently suggested to contribute to chromosome translocations in chronic and acute leukemia [38]. Our results of chromosome breaks within the MAR/SAR sequence supported the role of chromatin structure in chromosome rearrangements.

Since EBV infection and *LMP1 *expression both resulted in apoptosis and DNA fragmentation [7,8,39], it is possible that during EBV infection, apoptosis is induced and resulted in chromosome breaks that lead to chromosome rearrangements in cells that survive apoptosis. A single event of infection may not be sufficient to initiate cancer, however, multiple cycles of infection or reactivation and latency would increase the possibility of tumorigenesis by increasing the number of chromosome anomalies. This notion is supported by a study reporting that recurrent chemical reactivations of EBV promotes genome instability as well as enhances tumor progression of nasopharyngeal carcinoma cells [40].

## Conclusions

High cell density and *LMP1 *expression induced apoptosis in NPC cells and subsequently resulted in *MLL *bcr cleavage at the MAR/SAR region. This cleavage is most likely mediated predominantly by CAD and partially by other nucleases. Since *MLL *locates at 11q23, a common deletion site in NPC, our results suggest a possibility of stress- or virus-induced apoptosis in the initiation of chromosome rearrangements at 11q23, where the chromatin structure plays a role in defining the site of chromosome rearrangement. These results tally with findings in leukemia, suggesting a possible common mechanism of chromosome rearrangement in different cancer types.

## Competing interests

The authors declare that they have no competing interests.

## Authors' contributions

SPS contributes to the main idea of the project, the design of the study, interpretation of data and writing of manuscript. PHCY have been involved in the detailed experimental design, acquisition of data, interpretation of data and analysis. All authors read and approved the final manuscript.
